# Supplementary figures and images for: Microbial Signatures in COVID-19: Distinguishing Mild and Severe Disease via Gut Microbiota
Source: Biomedicines. 2024 May 1;12(5):996. doi: 10.3390/biomedicines12050996 (PMC11118803; doi:10.3390/biomedicines12050996)

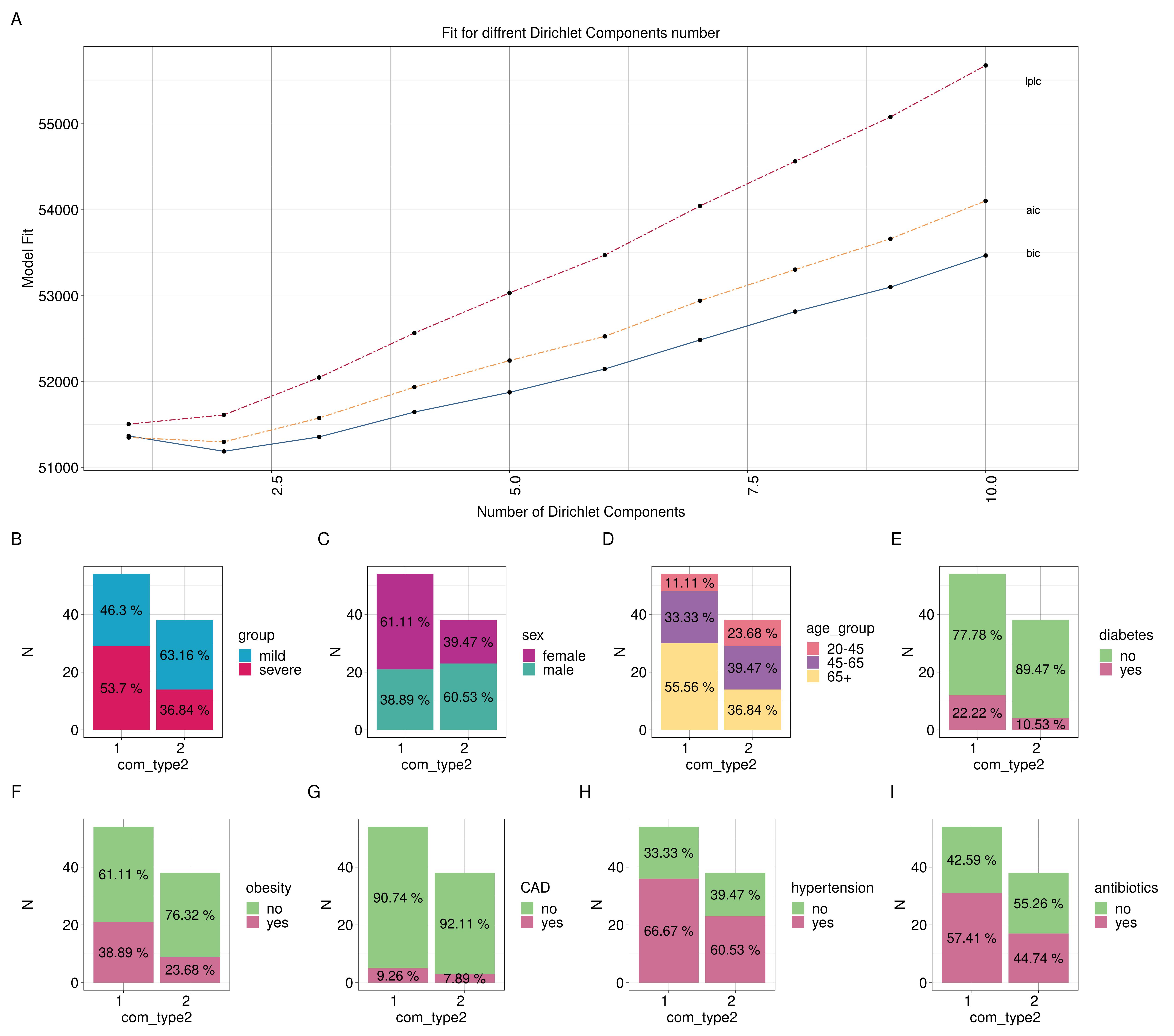

Supplement: Supplementary file 1 [file biomedicines-12-00996-s001.zip › S2_Fig.png]

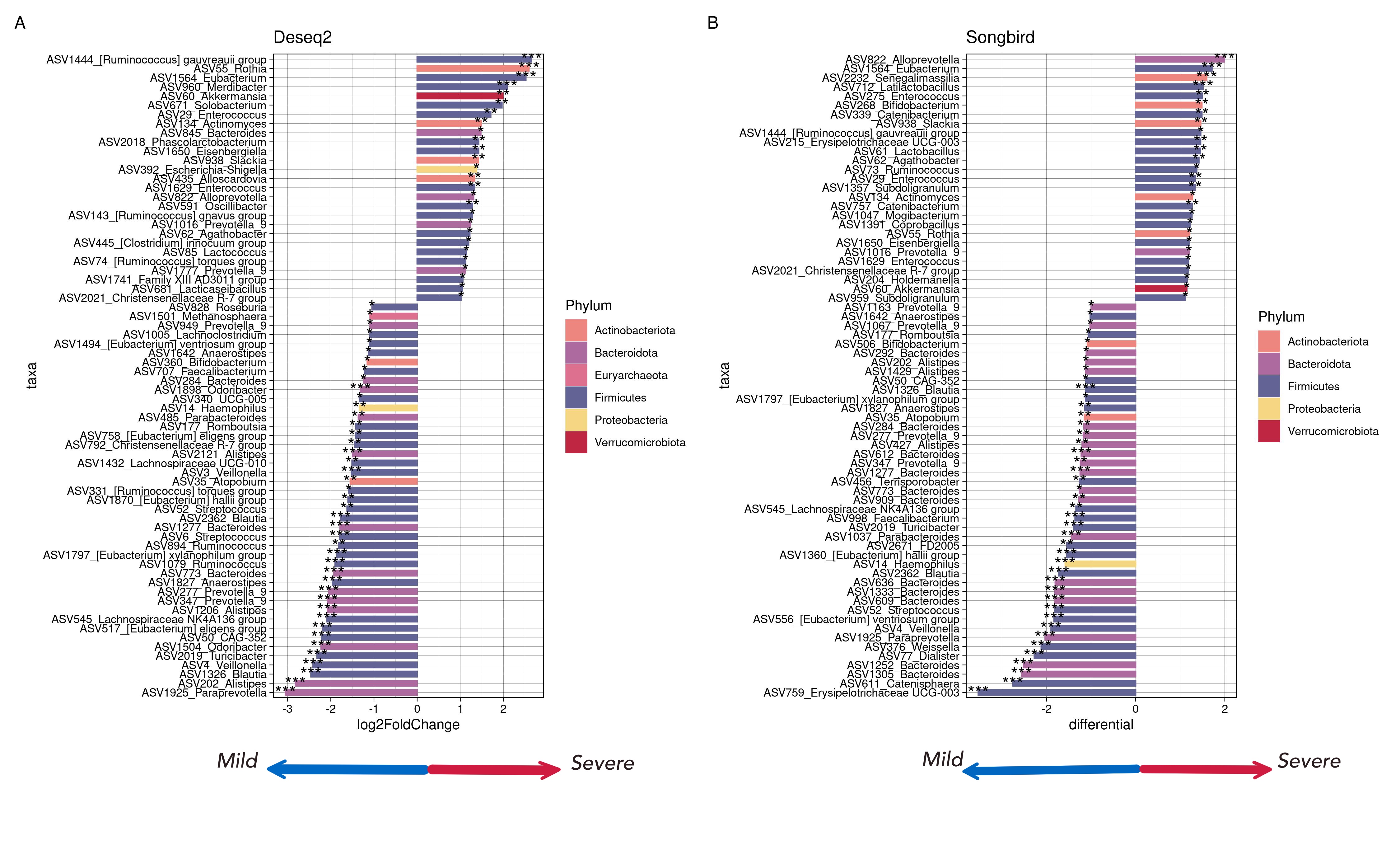

Supplement: Supplementary file 1 [file biomedicines-12-00996-s001.zip › S3_Fig.png]

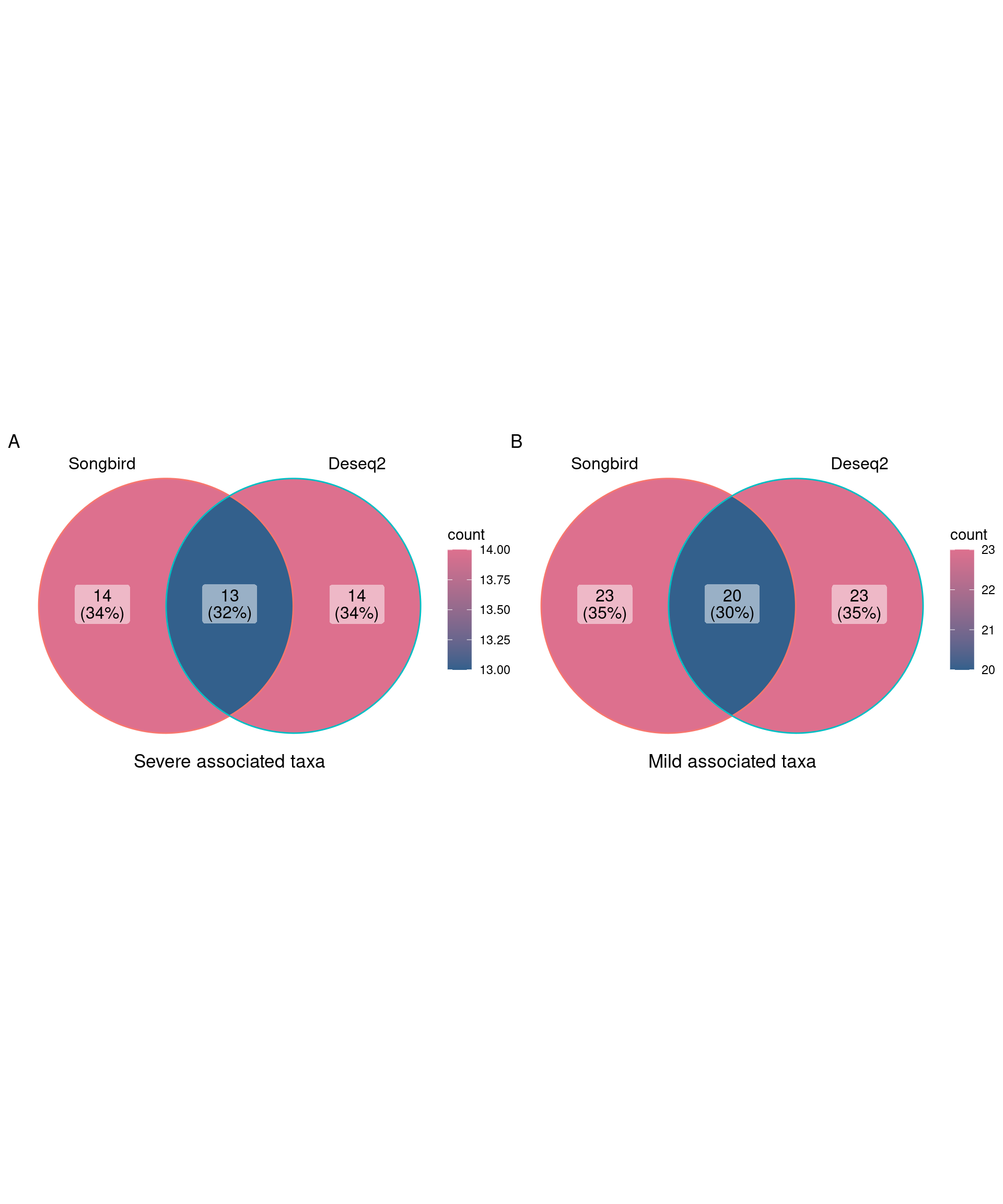

Supplement: Supplementary file 1 [file biomedicines-12-00996-s001.zip › S4_Fig.png]
